# Analysis of serum antioxidant capacity and gut microbiota in calves at different growth stages in Tibet

**DOI:** 10.3389/fmicb.2022.1089488

**Published:** 2023-01-30

**Authors:** Xinyu Zhang, Zhijun Cao, Hongjian Yang, Yajing Wang, Wei Wang, Shengli Li

**Affiliations:** State Key Laboratory of Animal Nutrition, College of Animal Science and Technology, China Agricultural University, Beijing, China

**Keywords:** gut microbiota, Holstein calf, high altitude, antioxidant capacity, different growth stages

## Abstract

**Introduction:**

The hypoxic environment at high altitudes poses a major physiological challenge to animals, especially young animals, as it disturbs the redox state and induces intestinal dysbiosis. Information about its effects on Holstein calves is limited.

**Methods:**

Here, serum biochemical indices and next-generation sequencing were used to explore serum antioxidant capacity, fecal fermentation performance, and fecal microbiota in Holstein calves aged 1, 2, 3, 4, 5, and 6 months in Tibet.

**Results and Discussion:**

Serum antioxidant capacity changed with age, with the catalase and malondialdehyde levels significantly decreasing (*p* < 0.05), and superoxide dismutase levels significantly increasing (*p* < 0.05) with age. No significant differences (*p* > 0.05) in total volatile fatty acid levels were noted between the groups. In all groups, Firmicutes, Bacteroidetes, and Actinobacteria were the three most dominant phyla in the gut. Gut microbial alpha diversity significantly increased (*p* < 0.05) with age. Principal coordinate analysis plot based on Bray–Curtis dissimilarity revealed significant differences (*p* = 0.001) among the groups. Furthermore, the relative abundance of various genera changed dynamically with age, and the serum antioxidant capacity was associated with certain gut bacteria. The study provides novel insights for feeding Holstein calves in high-altitude regions.

## 1. Introduction

The Tibetan Plateau, the world’s highest plateau (Gu et al., 2007), occupies a quarter of the Chinese land area (Wu, 2001). Its unique environment and climate are characterized by low temperature, strong ultraviolet radiation, and low atmospheric partial oxygen pressure (Guo et al., 2014). This poses a major challenge to local animals (Cheviron and Brumfield, 2012; Guo et al., 2014). Hypoxia causes inflammation, which affects immune function, leading to altitude sickness and chronic diseases, such as pulmonary hypertension (Pham et al., 2021). Furthermore, hypoxic conditions affect redox homeostasis (Samanta and Semenza, 2017). Hypobaric hypoxia negatively affects the body’s redox reactions, leading to the production of reactive species, which disrupt cellular components (such as lipids and proteins), leading to degradation of the antioxidant system (Strapazzon et al., 2016; Mrakic-Sposta et al., 2021). In addition, oxidative stress contributes to the development of inflammation, termed oxidative inflammation, which may affect the animal’s health.

Calves are important to Tibetan economy. Calf development is considered one of the most important issues affecting the health and profitability of dairy cow (Soberon et al., 2012). Calf growth and development are influenced by energy intake and genetic potential (Lohakare et al., 2012). Calves are functional monogastric animals at birth, and their nutrient and energy intake mainly depend on the intake of milk or milk replacer until the rumen function matures (Khan et al., 2016; Steele et al., 2016; Korst et al., 2017).

The intestinal microbiota is considered an endocrine organ, and the molecules produced by the gut microbes affect the host’s health. Intestinal microbiota is critical for the development and differentiation of the gut mucosal epithelium, as well as the mucosal immune system (Sommer and Bäckhed, 2013). Hence, it plays a key role in immune system stimulation, and metabolic and nutritional homeostasis (Ayalew et al., 2021). In addition, colonization of young ruminants, such as goat (Zhuang et al., 2020), yak (Guo et al., 2020), and pig (Ma et al., 2019), by gut microorganisms affects the production performance and lifelong health of these ruminants. Guo et al. (2020) reported the development and maturation of rumen microbiome throughout the life of yak bred in the highlands. However, there are only a few reports on the establishment of gut microbiota in dairy cow in high-altitude regions during early animal development. Furthermore, the relationship between serum antioxidant capacity and gut microbiota in calves in high-altitude regions is not known.

In the current study, we analyzed the serum biochemical indices and fecal microbiota in Holstein calves (*holsatia*) bred in Tibet, aged from 1 month to 6 months, to address the above-mentioned knowledge gaps. We hypothesized that the serum antioxidant capacity and gut microbiota would change with age and that the gut microbiota contributes to serum antioxidant capacity at high-altitude regions. Hence, we compared the serum antioxidant capacity, fecal fermentation performance, and gut microbiota in Holstein calves of different ages grow in high-altitude regions to explore the resistance mechanism to high-altitude hypoxic environments in calves. To our knowledge, this is the first study on the serum antioxidant capacity and gut microbiota in Holstein calves at a high altitude; furthermore, it provides new insights into the growth and development of ruminants at high altitudes.

## 2. Materials and methods

The study procedures were approved by the Ethical Committee of China Agricultural University’s College of Animal Science and Technology (permit number: AW22121202-1-2).

### 2.1. Study region, animals, and management

In August 2021, 36 male Holstein calves were selected according to age from a herd of 365 calves that were born in 2021, had the same father, and were raised at Zhizhao Farm (Lhasa, China). Calves with a similar body weight at birth (35.29 ± 1.67 kg) were divided into six groups (1, 2, 3, 4, 5, or 6 months of age; M1, M2, M3, M4, M5, and M6 groups, respectively) were selected, with six individuals per group. Calves in the M1 and M2 groups were maintained in individual pens (3.0 m × 1.6 m × 1.8 m; length × width × height). To keep the pens clean and dry, oat hay was used as bedding, and it was replaced daily. The calves were fed milk three times daily (08:30, 14:30, and 19:30 h) before weaning; the diet was supplemented with starter from 7 days of age. The calves were weaned gradually at 3 months of age. After weaning, the calves are fed oat hay and the starter *ad libitum*. The composition of the starter was as follows: crude protein, 24.08%; ether extract, 4.50%; ash, 11.52%; calcium, 1.42%; and phosphorus, 0.7% (dry matter based).

### 2.2. Blood sample collection and analysis

For analysis, 15 ml of blood was collected from the tail root using vacuum tubes before morning feeding. The blood samples were immediately centrifuged for 10 min at 3000 rpm. The obtained serum was immediately placed and stored at −20°C for testing. Tumor necrosis factor α (TNF-α), immunoglobulins (Ig)A, G (IgG), and M (IgM) were analyzed using enzyme-linked immunosorbent assay kits (Laibotairui Bioengineering Institute, Beijing, China). The GF-D200 automatic biochemical analyzer (Jiangsu Zecheng Bioengineering Institute, CLS880, Jiangyin, China) was used to determine serum aspartate transaminase (AST), alanine transaminase (ALT), and total cholesterol (TC) levels. Commercial kits (Nanjing Jian Cheng Bioengineering Institute, Nanjing, China) were used to determine the total antioxidant capacity (T-AOC), and superoxide dismutase (SOD), glutathione peroxidase (GSH-Px), malondialdehyde (MDA), and catalase (CAT) levels in the serum. All assays were performed according to the manufacturers’ guidelines.

### 2.3. Fecal sample collection

Some fecal samples from Holstein calves were immediately frozen in liquid nitrogen (−196°C) for fecal microbiota analysis and the remaining samples were stored at −20°C for analyzing fecal fermentation parameters.

### 2.4. Volatile fatty acid (VFA) analysis

For VFA analysis, after dilution, the feces were thawed and centrifuged at 8000 × *g* for 15 min at 4°C. VFAs in the supernatant were determined using gas chromatography, as described elsewhere (Erwin et al., 1961).

### 2.5. DNA extraction, polymerase chain reaction (PCR), and 16S rRNA sequencing

Bacterial DNA was extracted from 1 g of fecal sample using the OMEGA kit (Omega Bio-Tek, Norcross, GA, USA), following the manufacturer’s instructions. DNA concentration and purity were evaluated using the Nanodrop 2000 Spectrophotometer (Thermo Scientific, Waltham, USA). To amplify the V3–V4 region of the bacterial 16S rRNA gene, primers 338F (5′-ACTCCTACGGGAGGCAGCA-3′, forward) and 806R (3′-GGACTACNNGGGTATCTAAT-5′, reverse) were used. The following PCR amplification program was used: 5-min denaturation at 95°C; 28 cycles at 95°C for 45 s, 55°C for 50 s, and 72°C for 45 s; and a final extension at 72°C for 10 min. The amplified fragments were identified using 2% (w/v) agarose gel electrophoresis, purified using the Agencourt AMPure XP kit (Beckman Coulter Genomics, Indianapolis, IN, USA), and quantified using PCR (ABI 9700; Thermo Fisher Scientific, Waltham, MA, USA). The purified PCR products were sequenced following standard protocols, using a 2 × 250 bp sequencing kit and Illumina MiSeq (Illumina, San Diego, CA, USA).

### 2.6. Quality control and sequence analysis

QIIME 1.8 (Caporaso et al., 2010) was used to filter out reads with scores ≤20 (low quality) and reads <200 bp, and to remove barcode tags. PEAR 0.9.6 (Zhang et al., 2014) was used to combine the sequences and Flash 1.20 (Magoč and Salzberg, 2011) was used to demultiplex them. UCHIME (UCHIME Algorithm) (Edgar et al., 2011) was used to eliminate reads and chimeric sequences with a combined length of less than 230 bp. All sequences were subsampled according to the same sample size for further analysis to eliminate errors. Ribosomal Database Project classifier (Cole et al., 2009) was used to classify the sequences into operational taxonomic units (OTUs) based on a sequence similarity threshold of 97%. OTUs were compared with those in the SILVA 128 database for bacterial species categorization (Quast et al., 2013). All values were obtained using UCLUST to generate a representative OTU table (Edgar, 2010).

QIIME 1.8 (Caporaso et al., 2010) was used to determine alpha diversity at the OTU level, including Chao, Shannon, Simpson, and Ace indices, and the results were plotted using “ggplot2” in R (version 4.0.5) (Wickham, 2009). For beta-diversity analysis, a Bray–Curtis dissimilarity matrix was used for principal coordinate analysis (PCoA) in R 4.0.5 using the “vegan” package (Oksanen et al., 2016). Functional differences in the fecal microbiota in samples were predicted using PICRUST 2 and two regions.^1^

### 2.7. Statistical analysis

Wilcoxon rank test was used to compare alpha-diversity indices among different groups using the “dplyr” package in R (Wickham, 2017). The Bray–Curtis dissimilarity matrices were analyzed in R, and subsequently, the PCoA analysis was performed; the results were displayed using the “ggplot2” tool. Kruskal–Wallis H test in R 4.0.5 was used to evaluate the difference in relative abundance at the phylum, family, and genus levels, as well as the microbiota function, among the six groups. The relationship among the core OTUs, age, and fecal fermentation parameters was analyzed and visualized using the “Psych” package (Revelle, 2018) and the “corrplot” package (Jami et al., 2013) in R.

## 3. Results

### 3.1. Effects of growth stage on serum biochemical indices

#### 3.1.1. Effects of growth stage on serum antioxidant capacity

**Figure 1:**
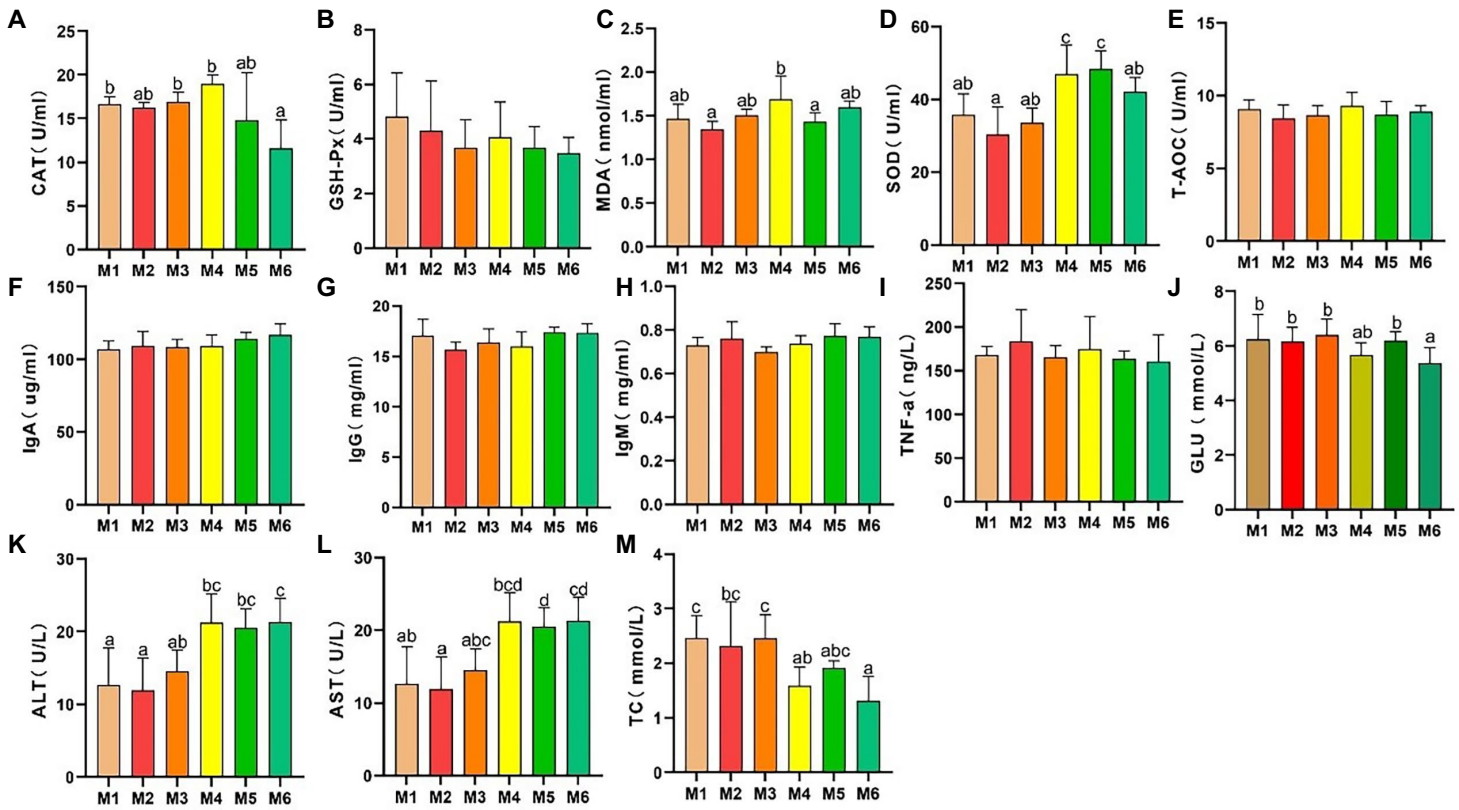
Effects of growth stage on serum antioxidant capacity and other biochemical indices in calves in Tibet. The following parameters were analyzed: **(A)** catalase (CAT) level; **(B)** glutathione peroxidase (GSH-Px) level; **(C)** malondialdehyde (MDA) level; **(D)** superoxide dismutase (SOD) levels; **(E)** total antioxidant capacity (T-AOC); **(F)** immunoglobulin (Ig) A level; **(G)** IgG level; **(H)** IgM level; **(I)** tumor necrosis factor α (TNF-α) level; **(J)** glucose (GLU) level; **(K)** alanine transaminase (ALT) level; **(L)** aspartate transaminase (AST) level; and **(M)** total cholesterol (TC) level. The results are shown as mean *±* SEM. The differences among the six groups are indicated by various letters (*p* < 0.05); the number of calves in each of the six groups was 6.

The results of serum antioxidant capacity analyses are presented in Figure 1. CAT activity (Figure 1A) decreased significantly (*p* < 0.05), whereas there were no significant differences (*p* > 0.05) in GSH-Px activity (Figure 1B) and T-AOC (Figure 1E) among the calf groups. However, MDA level (Figure 1C) exhibited volatility, which is associated with dietary changes. In addition, SOD activity (Figure 1D) increased significantly with age (*p* < 0.05).

#### 3.1.2. Effects of growth stage on other serum biochemical indices

As shown in Figures 1F–M, no significant differences in the serum IgA, IgG, IgM, and TNF-α levels were detected among the groups, indicating a lack of inflammation. In addition, the ALT and AST levels significantly increased (*p* < 0.05) with age, and with a major shift between the M3 and M4 growth stages. Furthermore, the serum glucose (GLU) and TC levels decreased with age.

### 3.2. Feces fermentation parameters of Holstein calves of different ages

**Figure 2:**
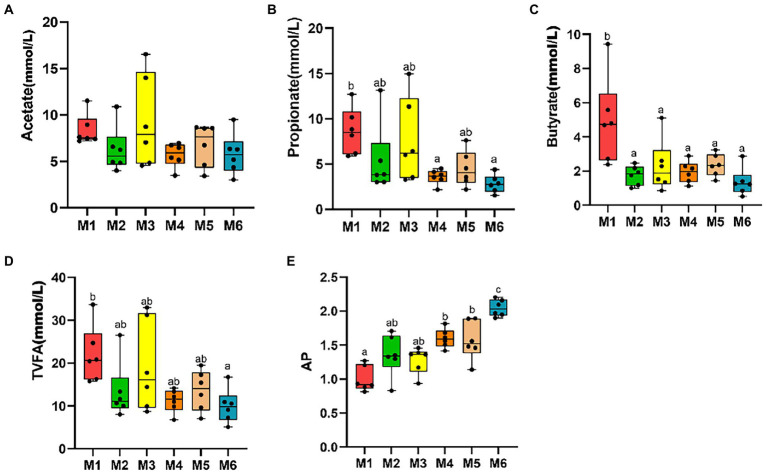
Effects of growth stage on fecal levels of volatile fatty acids (VFAs) in dairy calves. The following are shown in the figures: fecal levels of individual VFAs **(A–C)**, total volatile fatty acids (TVFA; **D**), and acetate to propionate ratio (AP; **E**). The results are presented as mean *±* SEM. The differences among the six groups are represented by various letters (*p* < 0.05); the number of calves in each of the six groups was 6.

No significant differences were observed among the six groups in terms of fecal levels of TVFA, acetate, propionate, butyrate, and valerate (*p* > 0.05), with the acetate to propionate ratio (AP) increasing with age (*p* < 0.05; Figure 2).

### 3.3. Gut microbiota of Holstein calves of different ages

#### 3.3.1. Sequencing metrics of the gut microbiota

Overall, 1,164,140 raw sequences were obtained, with an average of 34,260.78 ± 2992.40 (mean ± SD) in each sample. Furthermore, an average of 405.61 ± 161.85 OTUs per sample was detected at 3% sequence dissimilarity. In addition, the average Good’s coverage was 0.997 across all 36samples, implying adequate sequence coverage in the samples.

#### 3.3.2. Gut microbiota profiles

**Figure 3:**
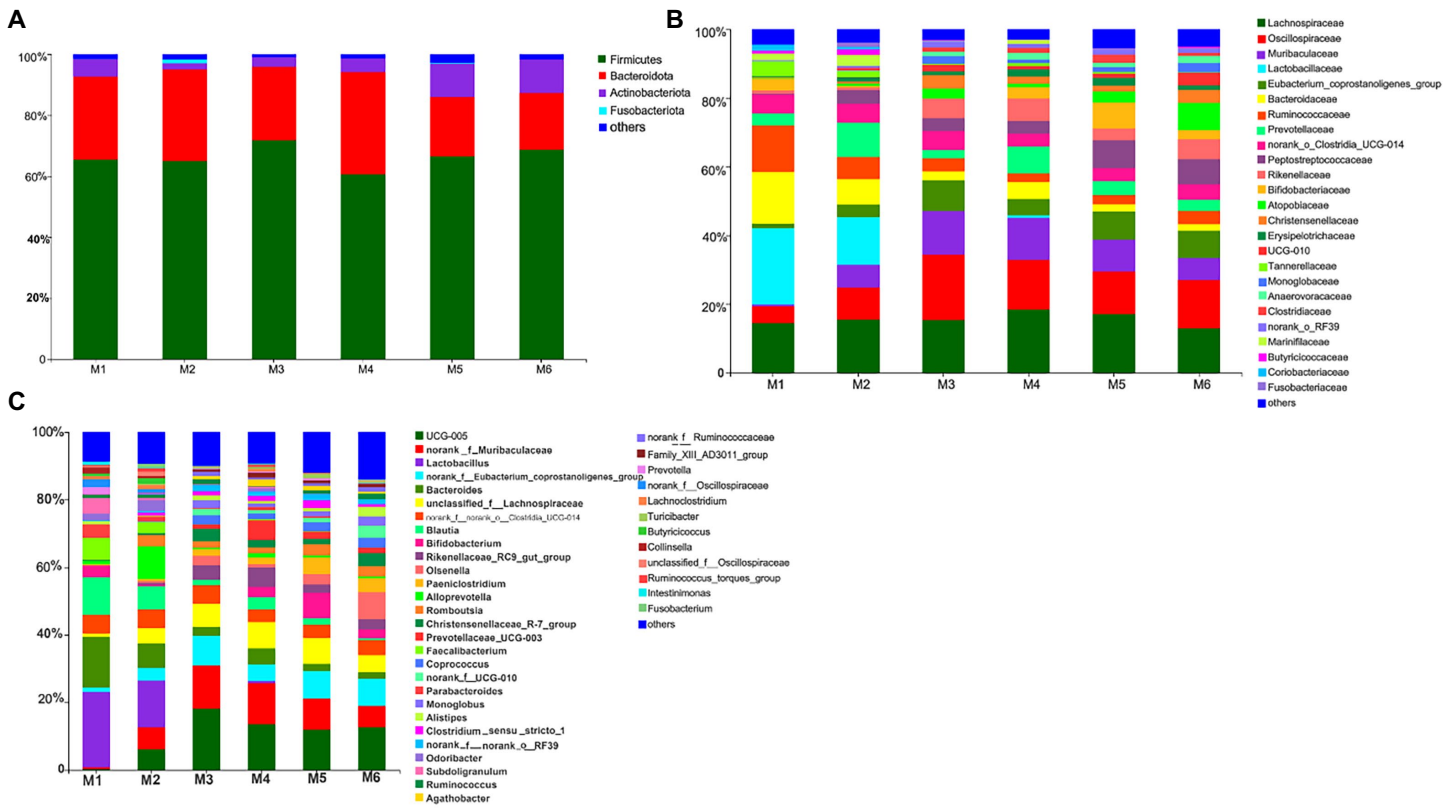
Composition of the fecal bacteria at the phylum **(A)**, family **(B)**, and genus levels **(C)** in different-age calves in Tibet. Others, bacteria with relative abundance ≤0.01%; the number of calves in each of the six groups was 6.

We identified 280 genera belonging to 100 families representing 16 phyla. As shown in Figure 3, at the phylum level, seven bacterial phyla present in all six groups and with relative abundance >0.01% were detected. The most dominant phyla were Firmicutes (65.94%), Bacteroidetes (29.34%), and Actinobacteria (4.03%), followed by Proteobacteria (0.31%), Cyanobacteria (0.14%), Spirochaetota (0.13%), and Patescibacteria (0.12%). At the family level, nine families present in all six groups and with relative abundance >5% were detected. The most dominant families were *Lachnospiraceae* (15.58%), *Oscillospiraceae* (12.41%), and *Muribaculaceae* (7.95%), followed by *Lactobacillaceae* (6.16%), Eubacterium_coprostanoligenes_group (5.78%), *Bacteroidaceae* (5.65%), *Ruminococcaceae* (5.49%), and *Prevotellaceae* (5.17%). Furthermore, six bacterial genera present in all groups and with relative abundance >5% were detected. The top three most abundant genera were *Ruminococcaceae_UCG-005* (10.39%), *norank_f__Muribaculaceae* (7.95%), and *Lactobacillus* (6.16%), followed by *norank_f__Eubacterium_coprostanoligenes_group* (5.78%), *Bacteroides* (5.65%), and *unclassified_f__Lachnospiraceae* (5.50%).

#### 3.3.3. Diversity of fecal microbiota

**Figure 4:**
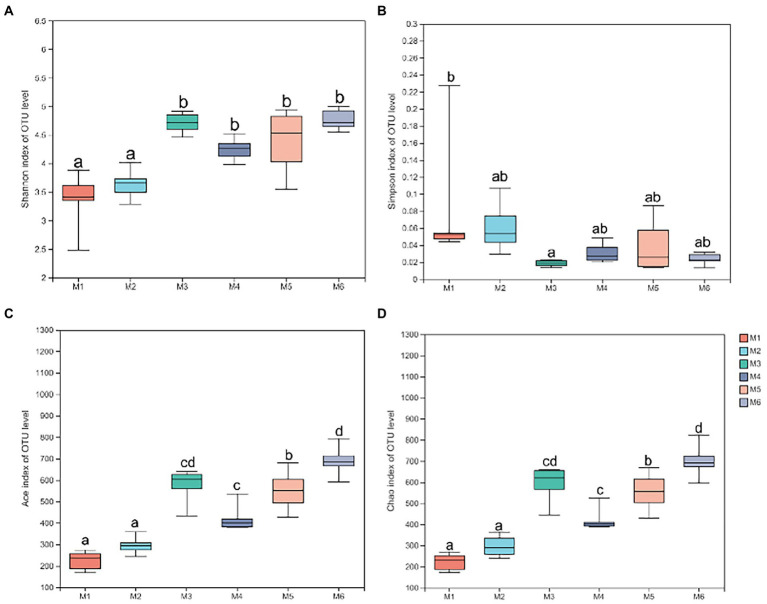
Alpha diversity analysis. **(A)** Shannon index at the operational taxonomic unit (OTU) level. **(B)** Simpson index at the OTU level. **(C)** Ace index at the OTU level. **(D)** Chao index at the OTU level. Data are shown as mean ± SEM. Different lowercase letters indicate significant differences among different groups (*p* < 0.05); the number of calves in each of the six groups was 6.

**Figure 5:**
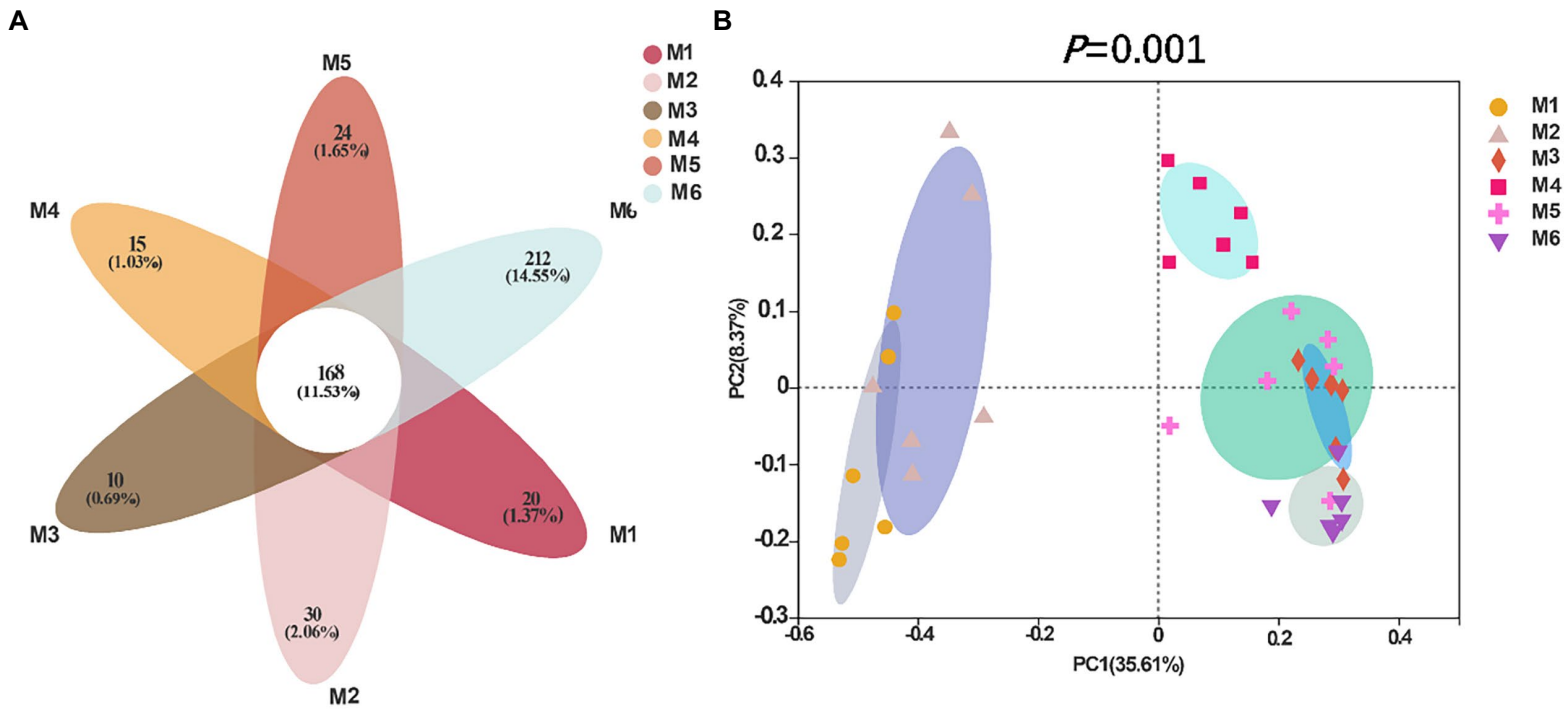
Flower diagram plot and beta-diversity analysis of the fecal microbiota of calves in different age groups. **(A)** Flower diagram plot. OTUs present in all groups are identified as the core community of all groups. **(B)** Principal coordinates analysis (PCoA) plots of microbiota in different samples; the number of calves in each of the six groups was 6.

Shannon (Figure 4A), Simpson (Figure 4B), Ace (Figure 4C), and Chao richness indices (Figure 4D) in calves at different growth stages were significantly different (*p* < 0.05). We evaluated the core bacteria in all calves and found 168 OTUs that were common in all samples (Figure 5A). To evaluate the presence of variations in the fecal microbiota in calves at different growth stages, we visualized the outcomes of Bray–Curtis dissimilarity analysis using a PCoA plot (Figure 5B). ANOSIM revealed that the six groups were statistically different (*p* = 0.001).

#### 3.3.4. Changes in the fecal bacteria in Holstein calves with age

We performed Kruskal–Wallis H test to determine significantly different genera (among the 10 most relatively abundant genera) between groups (Supplementary Image 1). With age, the relative abundance of *norank_f__Eubacterium_corostanoligenes_group* increased (*p* < 0.05); the relative abundance of *Ruminococcaceae_UCG-005*, *norank_f__Muribaculaceae* and *Rikenellaceae_RC9_gut_group, unclassified_f_Lachnospiraceae* increased and then decreased (*p* < 0.05); the relative abundance of *Lactobacillus, Bacteroides*, and *Blautia* decreased (*p* < 0.05); the relative abundance of *Olsenella* and *Bifidobacterium* increased overall (M1–M6 groups), but fluctuated after weaning (M4 group; *p* < 0.05).

#### 3.3.5. Correlation of fecal bacteria with fecal fermentative parameters in Holstein calves of different ages

We next investigated the potential effect of fecal bacteria on the fermentative parameters and serum antioxidant capacity in calves. Accordingly, we performed a correlation analysis between serum antioxidant capacity and VFAs, and the relative abundance of genera (top 50) using Spearman’s rank correlation (Supplementary Image 2). Considering the antioxidant capacity, 13 genera were significantly positively correlated (|*r*| > 0.3, *p* < 0.05) with the SOD level; 1 genus was positively associated with T-AOC; and 4 genera were positively correlated with the GSH-Px level. Furthermore, 24 genera were significantly correlated (*p* < 0.05) with the MDA level, of which 16 showed a positive correlation (*p* < 0.05) and 8 showed a negative correlation (*p* < 0.05). Seven genera were significantly correlated (*p* < 0.05) with the CAT level, of which 4 showed a positive correlation (*p* < 0.05) and 3 showed a negative correlation (*p* < 0.05). Considering VFAs, 3 genera significantly correlated (*p* < 0.05) with acetate levels, of which 2 showed a positive correlation (*p* < 0.05) and 1 showed a negative correlation (*p* < 0.05). Twelve genera were significantly correlated (*p* < 0.05) with the propionate level, of which 4 showed a positive correlation (*p* < 0.05) and 8 showed a negative correlation (*p* < 0.05). Furthermore, 13 genera significantly correlated (*p* < 0.05) with the butyrate level, of which 5 showed a positive correlation (*p* < 0.05) and 8 showed a negative correlation (*p* < 0.05). Finally, 10 genera significantly correlated (*p* < 0.05) with TVFAs, of which 5 showed a positive correlation (*p* < 0.05) and 5 showed a negative correlation (*p* < 0.05).

### 3.4. Functional predictions using PICRUSt2

The function of gut bacteria in dairy calves of different ages was predicted using PICRUST2 and differences in Kyoto Encyclopedia of Genes and Genomes (KEGG) pathway abundance among different groups were determined (Table 1). ANOSIM revealed the enrichment of six pathways in the six groups (*p* < 0.05; Table 1), with “biosynthesis of amino acids,” “microbial metabolism in diverse environments,” “purine metabolism,” “pyrimidine metabolism,” “amino sugar and nucleotide sugar metabolism,” and “glycolysis/gluconeogenesis” belonging to “Global and overview maps,” “Nucleotide metabolism,” and “Carbohydrate metabolism,” and all belonging to “Metabolism.”

**Table 1:** Functional predictions of significantly different KEGG pathways of fecal bacteria at three levels (only level III pathways that were significantly different at *p* < 0.05 and abundances >0.01% are shown).

KEGG_Pathway	Group						SEM	*p*
Level I/Level II/Level III	M1	M2	M3	M4	M5	M6		
*Metabolism*	0.769	0.769	0.764	0.771	0.764	0.765	0.0175	0.121
**Global and overview maps**	0.405	0.402	0.406	0.406	0.404	0.406	0.00063	0.156
Biosynthesis of secondary metabolites	0.089	0.089	0.091	0.091	0.091	0.091	0.0002	0.092
Biosynthesis of amino acids	0.043^bc^	0.043^c^	0.046^a^	0.045^abc^	0.045^abc^	0.046^ab^	0.0004	0.010
Microbial metabolism in diverse environments	0.045^a^	0.044^ab^	0.043^b^	0.043^b^	0.043^b^	0.043^b^	0.0001	0.006
**Nucleotide metabolism**	0.031^a^	0.030^a^	0.028^b^	0.029^ab^	0.029^ab^	0.028^ab^	0.0003	0.002
Purine metabolism	0.017^a^	0.017^ab^	0.015^c^	0.016^abc^	0.016^abc^	0.016^bc^	0.0002	0.001
Pyrimidine metabolism	0.013	0.013	0.012	0.012	0.013	0.012	8.32E-05	0.054
**Carbohydrate metabolism**	0.105	0.102	0.098	0.100	0.100	0.099	0.0006	0.130
Amino sugar and nucleotide sugar metabolism	0.013	0.012	0.011	0.012	0.012	0.011	0.0001	0.003
Glycolysis / Gluconeogenesis	0.013^a^	0.013^ab^	0.011^b^	0.012^ab^	0.012^b^	0.011^b^	0.0002	0.000
*Genetic information processing*	0.087	0.089	0.089	0.087	0.001	0.090	0.0020	0.522
**Translation**	0.0370	0.038	0.038	0.037	0.038	0.039	0.0002	0.084
Ribosome	0.024	0.025	0.025	0.025	0.025	0.026	0.0001	0.056
*Environmental information processing*	0.056	0.055	0.055	0.053	0.001	0.054	0.0195	0.774
**Cellular community—prokaryotes**	0.019^c^	0.020^bc^	0.022^a^	0.020^abc^	0.022^a^	0.021^ab^	0.0002	0.001
Quorum sensing	0.013	0.013	0.015	0.014	0.015	0.014	0.0002	0.006

Differences between four groups are presented in the form of different letter (*p*<0.05).

## 4. Discussion

As one of the extreme environments, high altitude poses a major challenge to animal survival. Thegut microbiota is important for the health of ruminants. Understanding the establishment of this microbial community and its changes with the host’s age is essential for understanding the core microbial community and its effect on the host (Ley et al., 2008; Zilber-Rosenberg and Rosenberg, 2008; Jami et al., 2013). To date, some studies have focused on the gastrointestinal microbes of young indigenous ruminants, such as yak (Guo et al., 2020), bred at a high altitude, but no such data are available for Holstein dairy calf. Therefore, we explored the dynamic changes in fecal microbiota and serum antioxidant capacity in the early growth stages of Holstein calves in Tibet (from 1 month to 6 months of age).

First, we determined the serum antioxidant capacity and other serum biochemical indices. The analysis suggested that the serum antioxidant capacity (including CAT, MDA, and SOD levels) changes between the M3 and M4 growth stages. We interpreted this finding to indicate oxidative stress experienced by calves because of weaning stress. Unlike the early weaning practiced at 2 months of age in most areas, the early weaning at high altitudes occurs when the calf is 3 months old because of poor living conditions. Weaning stress induces oxidative damage, as reported previously (Luo et al., 2016; Wei et al., 2017). Weaning of high-altitude calves results in dual stress of weaning and hypoxia. The antioxidant defense system plays an important role under extreme stress, and alterations in the CAT, MDA, and SOD levels reveal systemic oxidative damage. Hence, weaning led to oxidative injury and altered antioxidant enzyme activities. ALT and AST are important indicators of liver function, and changes in the AST and ALT levels indicate that oxidative stress may lead to liver cell damage (Benerji, 2013). In fact, oxidative stress is a common mechanism damaging hepatocellular function.

We also detected fluctuations in the serum GLU and fecal VFA levels (including acetate, propionate, butyrate, TVFAs, and acetate to propylene ratio) in Holstein calves before and after weaning, which may be associated with a shift in the way energy is supplied. As reported previously, a decrease in the serum GLU level with age may be caused by decreased milk provision (Hill et al., 2010). This indicates that the serum GLU level decreases with the development of ruminant function in calf (Hugi and Blum, 1997). After weaning, the energy in calf is mainly derived from VFAs produced *via* fermentation by intestinal microbiota, and it no longer solely depends on the intake of milk (Tao et al., 2018). In addition, the production of VFAs may be related to the structure of the intestinal microbiota. Therefore, we explored the differences in the gut microbiota in calves of different ages. Indeed, we observed significant differences according to age.

Between weaning and 1 year of age, the rumen of dairy cow contains adult-like microbiota (Dill-McFarland et al., 2017). Age-related differences in the gut bacteria have also been observed in dairy calf. Particularly, weaning stress induces disturbances in the gut microbiota in calf (Kim et al., 2012; Devine et al., 2013) In the current study, we observed the greatest changes in microbiota between the M3 and M4 groups. The relative abundance of *Ruminococcaceae_UCG-005*, *norank_f_Muribacuiaceae*, *Lactobacillus*, *norank_f_Eubacterium_coprostanoligenes_group*, *Bacteroides*, *unclassified_f_Lachnospiraceae*, *Blautia*, *Bifidobacterium*, *Rikenellaceae_RC9_gut_group*, and *Olsenella* was affected by weaning. Among those, the relative abundance of *Ruminococcaceae_UCG-005* increased and stabilized after weaning. These bacteria are critical probiotics in the animal intestine, and degrade starch and cellulose by secreting copious amounts of cellulase and hemicellulase, with the degradation products providing energy to the host (Kim et al., 2012). *Ruminococcaceae_UCG-005* is strongly linked to chronic inflammation, metabolic disorders, and mycotoxin exposure in weaned pigs (Mateos et al., 2018). Furthermore, the relative abundance of *norank_f__Muribaculaceae* and *Rikenellaceae_RC9_gut_group* increased and then decreased. *Muribaculaceae* members are specialists in the fermentation of complex polysaccharides (Ormerod et al., 2016; Lagkouvardos et al., 2019) and produce propionate as a fermentation end product (Smith et al., 2021). *Rikenellaceae_RC9_gut_group* degrades cellulose and hemicellulose, and can produce propionate, acetate, and/or succinate as fermentation end products (Zened et al., 2013; Graf, 2014; Rosenberg et al., 2014; Sha et al., 2020).

We also observed that the relative abundance of *Lactobacillus* decreased and the genus ceased to be detectable after the M4 stage. *Lactobacillus* members exert a variety of beneficial effects in the host, including affecting antioxidant capacity (Lin and Chang, 2000; Shi et al., 2021). In fact, *Lactobacillus*, as probiotics, increases the serum SOD and GSH-Px levels (Martarelli et al., 2011). This suggests that a decrease in the relative abundance of *Lactobacillus* may decrease the antioxidant capacity in calves at high altitudes. Consistent with this, *Lactobacillus* and *Bifidobacterium* contribute to the increase in erythrocyte SOD and GSH-Px levels, and total antioxidant status (Ejtahed et al., 2012). Furthermore, the relative abundance of *norank_f__Eubacterium_corostanoligenes_group* increased in the M1–M3 growth period, then decreased, and increased again after the M4 period, which was associated with decreased TC, as previously reported (Madden, 1995). The relative abundance of *unclassified_f_Lachnospiraceae* also fluctuated. This bacterium is an important butyrate producer residing in the gut (Dahiya et al., 2019).

The relative abundance of *Bacteroides* and *Blautia* decreased significantly (fluctuating at M4). *Bacteroides* members are well-known for their ability to degrade polysaccharides (Lapébie et al., 2019). *Blautia* is a novel functional genus with potential probiotic components. The relative abundance of *Bifidobacterium* drastically decreased over the M1–M3 period, but then increased (and fluctuated) in M4–M6 calves. *Bifidobacterium* members are dominant bacteria that provide beneficial effectors to calves during the milk-feeding period Finally, the relative abundance of *Olsenella* increased overall (M1–M6) but fluctuated (Vlková et al., 2006) after weaning (the M4 group). *Olsenella* produces VFAs by fermenting starch and glycogen substrates (Göker et al., 2010).

Exposure to a hypoxic environment at a high altitude disrupts the systemic redox balance and leads to hypoxic oxidative stress (Samanta and Semenza, 2017; Gaur et al., 2021), which is related to the damage of the intestinal barrier (McKenna et al., 2022; Wang et al., 2022). In the current study, using Spearman correlation analysis, we analyzed the relationship between the gut microbiota and serum antioxidant capacity. Many bacteria were associated with the antioxidant capacity, including members of *Lachnospiraceae* and *Ruminococcaceae*. These findings suggest that supplementation of solid starters at weaning is the main cause of changes in the antioxidant status and gut microbiota during the growth and development of calves (Zhuang et al., 2020). Increasing the abundance of *Lachnospiraceae* and *Ruminococcaceae* members has been reported to control host oxidative stress in a previous study (Uchiyama et al., 2022), which is consistent with the findings of the current study.

Further studies are needed to explore the mechanism of oxidative damage in calves at high altitudes. The findings of the current study suggest that strategies that alter the abundance of certain bacteria, such as supplementation of antioxidant additives, could be used to regulate hypoxic stress and improve high-altitude adaptability of young animals.

## 5. Conclusion

In the current study, for the first time, we evaluated the serum antioxidant capacity and gut microbiota in Holstein calves at a high altitude. The analysis revealed that the serum antioxidant capacity and gut microbiota change with calf age. We observed that the gut microbiota in each age group change temporally, which was related to changes in the diet, growth development, and gut microbiota interactions. In addition, based on the correlation between serum antioxidant capacity and gut microbiota, we identified specific microbes that are related to the serum antioxidant capacity. This study provides new insights into how reshaping gut microbiota could improve the health and production performance of Holstein calves bred at high altitudes.

## Data availability statement

The datasets presented in this study can be found in the NCBI repository, accession number PRJNA851234.

## Ethics statement

The animal study was reviewed and approved by the Ethical Committee of China Agricultural University’s College of Animal Science and Technology (permit number: AW22121202-1-2). Written informed consent was obtained from the owners for the participation of their animals in this study.

## Author contributions

XZ performed the experiments and wrote the manuscript. WW, ZC, HY, YW, and SL reviewed and provided guidance for the manuscript and experiment. All authors contributed to the article and approved the submitted version.

## Funding

The services used in this study were funded by the Ministry of Agriculture and Rural Affairs of China: Experiment and Demonstration of Adaptive Production Technology for Dairy Cows in High Altitude Regions (16190319) and China Agriculture Research System of MOF and MARA (CARS36).

## Conflict of interest

The authors declare that the research was conducted in the absence of any commercial or financial relationships that could be construed as a potential conflict of interest.

## Publisher’s note

All claims expressed in this article are solely those of the authors and do not necessarily represent those of their affiliated organizations, or those of the publisher, the editors and the reviewers. Any product that may be evaluated in this article, or claim that may be made by its manufacturer, is not guaranteed or endorsed by the publisher.

## Footnotes

^1^ https://github.com/picrust/picrust2
