# Supplementary figures and images for: Analysis of serum antioxidant capacity and gut microbiota in calves at different growth stages in Tibet
Source: Front Microbiol. 2023 Jan 30;13:1089488. doi: 10.3389/fmicb.2022.1089488 (PMC9927023; doi:10.3389/fmicb.2022.1089488)

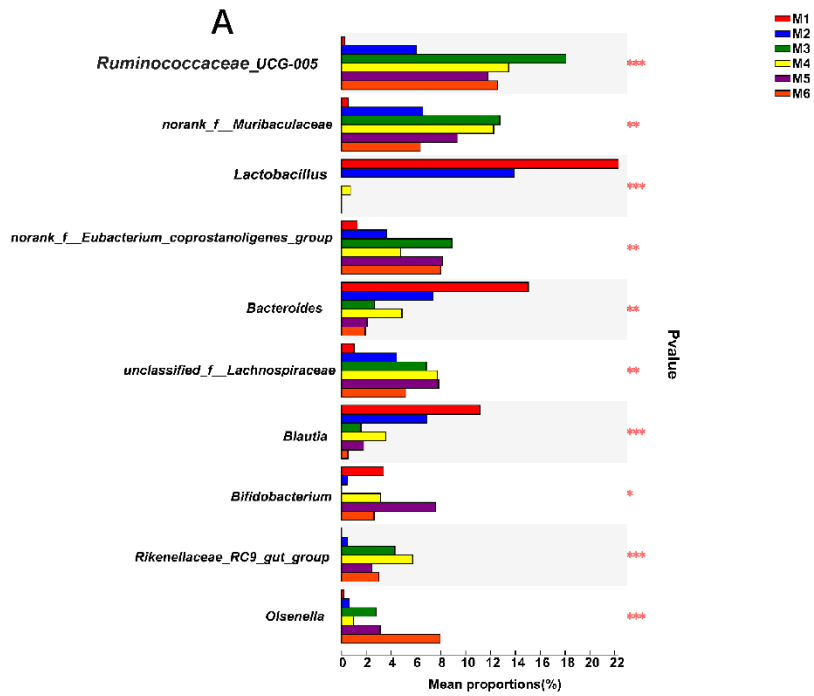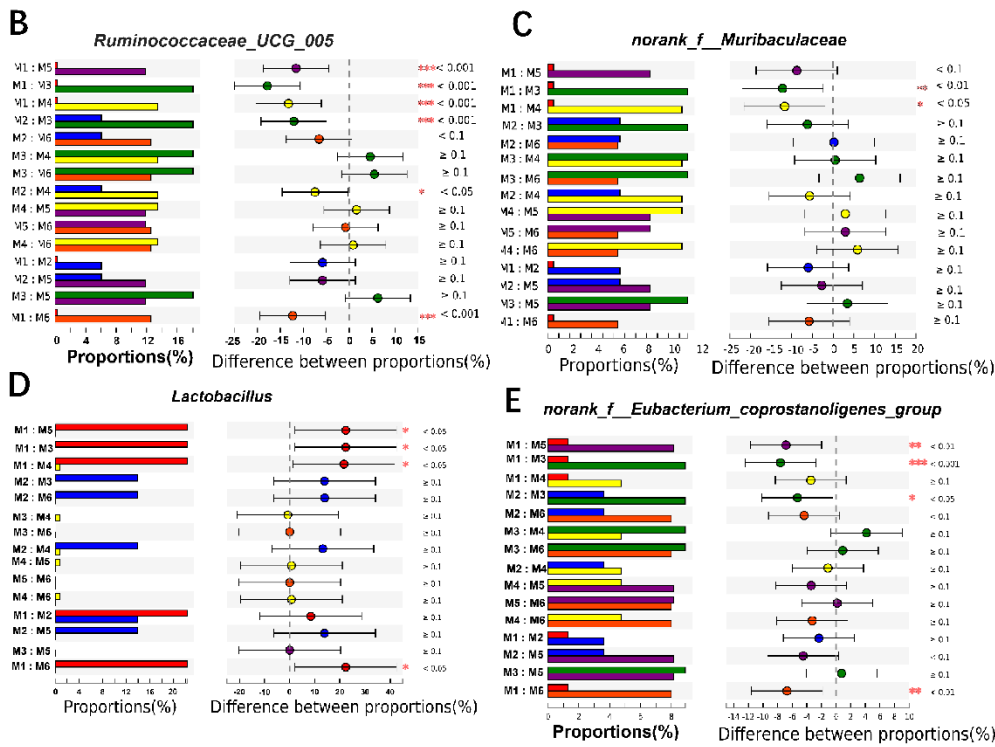

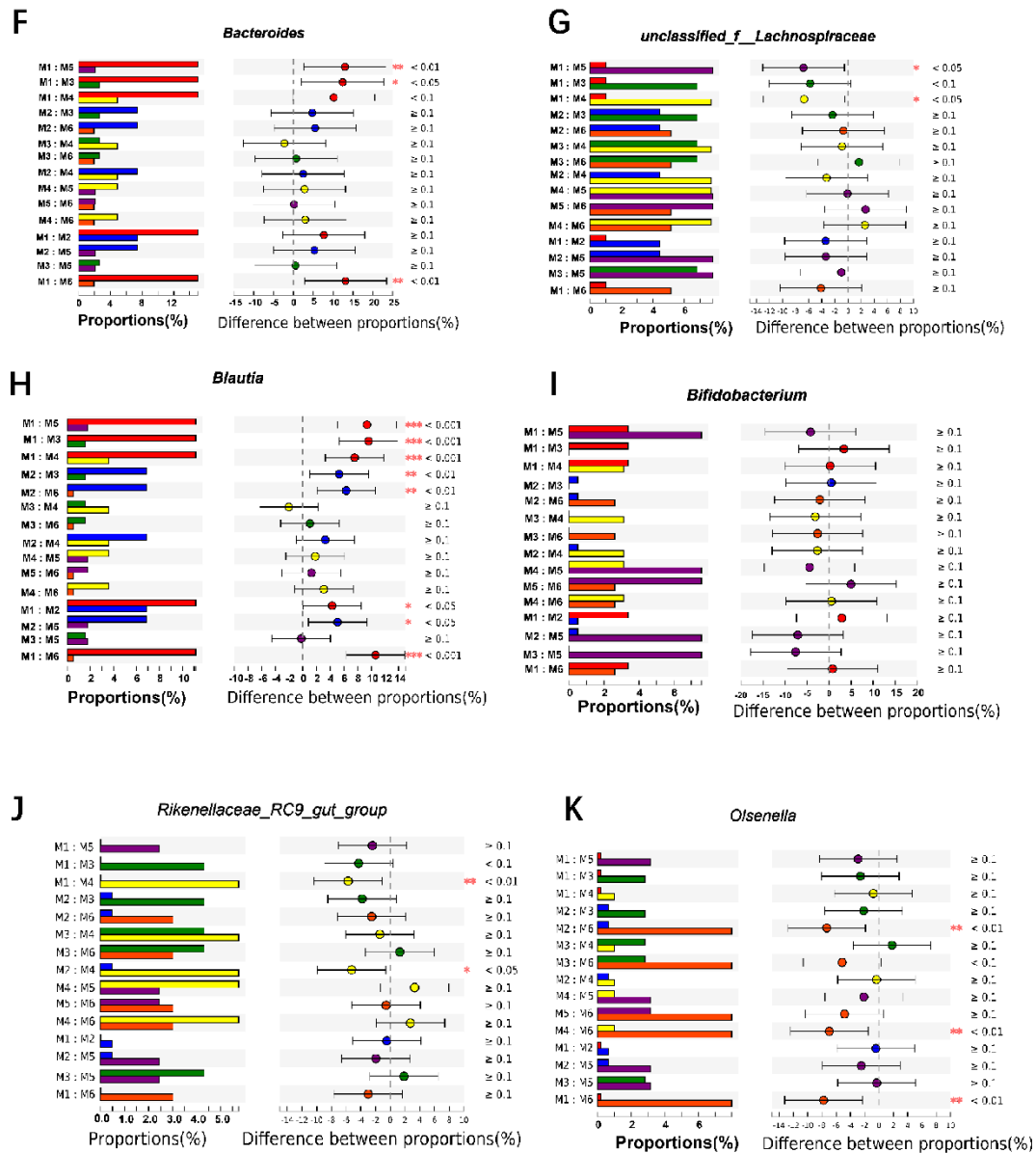

**Figure 6.**

Supplement: Supplementary file 1 [file Image_1.pdf]

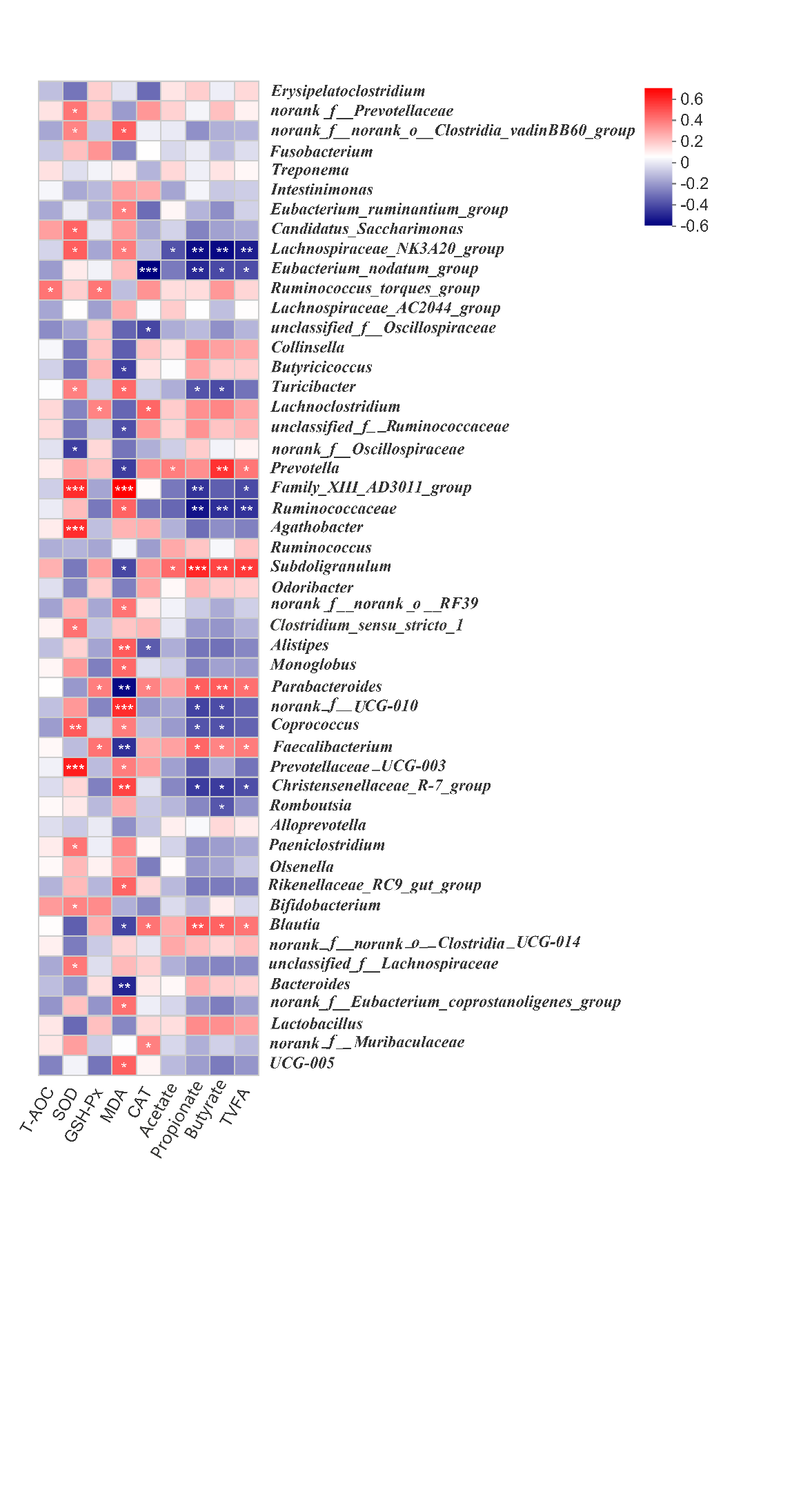

Supplement: Supplementary file 2 [file Image_2.png]
